# Exhausted PD-1^+^ TOX^+^ CD8^+^ T Cells Arise Only in Long-Term Experimental *Trypanosoma cruzi* Infection

**DOI:** 10.3389/fimmu.2022.866179

**Published:** 2022-06-03

**Authors:** Rosa Isela Gálvez, Thomas Jacobs

**Affiliations:** Protozoa Immunology, Bernhard Nocht Institute of Tropical Medicine, Hamburg, Germany

**Keywords:** *Trypanosoma cruzi*, co-inhibitory receptors, Chagas Disease, T cell exhaustion, TOX, TIM-3, PD-1

## Abstract

Infection with *Trypanosoma cruzi* remains the most important neglected zoonosis in Latin America. This infection does not lead to specific symptoms in the acute phase, but chronic infection can result in Chagas disease (CD) with cardiac and/or gastrointestinal manifestations that can lead to death. CD8^+^ T cells are highly effective and essential to control this infection, but fail to eliminate all parasites. In this study, we show that the CD8^+^ T cells are modulated by the transient induction of co-inhibitory receptors during acute infection of C57BL/6 mice. Therapeutic intervention strategies with blocking antibodies only had a marginal effect on the elimination of parasite reservoirs. Only long-term chronic infection gave rise to dysfunctional CD8^+^ T cells, which were characterized by high expression of the inhibitory receptor PD-1 and the co-expression of the transcription factor TOX, which plays a crucial role in the maintenance of the exhausted phenotype. PD-1^+^ TOX^+^ CD8^+^ T cells isolated from the site of infection produced significantly less IFN-γ, TNF-α and Granzyme B than their PD-1^-^ TOX^-^ CD8^+^ T cell counterparts after *T. cruzi*-specific stimulation *ex vivo*. Taken together, we provide evidence that, in the context of experimental infection of mice, the magnitude of the CD8^+^ T cell response in the acute phase is sufficient for parasite control and cannot be further increased by targeting co-inhibitory receptors. In contrast, persistent long-term chronic infection leads to an increase of exhausted T cells within the tissues of persistence. To our knowledge, this is the first description of infection-induced CD8^+^ T cells with an exhausted phenotype and reduced cytokine production in muscles of *T. cruzi*-infected mice.

## Introduction

Infection with the obligatory intracellular protozoan parasite *Trypanosoma cruzi* (*T. cruzi*) and Chagas disease (CD) remains an unsolved, ancient affliction in Latin America (LA) (1). It is estimated that more than 8 million people are infected and that 99% do not have access to either diagnosis or treatment (2, 3). Impoverished areas are particularly affected, with more than 65 million people facing the risk of infection every day (4). Migration out of LA has exacerbated CD expansion to other countries, which must now face this new emerging health problem, since 30-35 % of chronically infected cases will develop clinical cardiac or gastrointestinal forms of CD (5, 6). Accordingly, understanding immunity against *T. cruzi* is the key to developing new approaches, such as immune interventions or vaccines, to combat this harmful disease.

Although controlling an acute infection involves the complex interaction of soluble molecules, effector cells of the innate immune system and an adaptive response (7), an adequately regulated CD8^+^ T cell response is essential to successfully control the replication of the parasite over decades (8). Considering that *T. cruzi* parasites have developed complicated interaction mechanisms with mammalian hosts, there is still limited understanding of two crucial topics: How the parasite evades complete clearance through CD8^+^ T cells and which specific cellular immune regulatory processes prevent the onset of the disease over time. Similar to chronic viral infections, the persistence of *T. cruzi* parasites throughout the host’s lifespan leads to a prolonged effector T cell phase that can drive the cells to functional exhaustion, as is known to occur for anti-viral T cells. In different models of chronic viral infections and tumorigenesis, a hallmark of decreased T cell immunity is the co-expression of co-inhibitory receptors such as Programmed cell death 1 (PD-1), T-cell immunoglobulin mucin-3 (TIM-3), and Lymphocyte-activation gene 3 (LAG-3). Their inhibitory effect is characterized by a progressive loss of function and reduced production of effector cytokines (9–11). Strategies to restore the functionality of CD8^+^ T cells expressing co-inhibitory receptors involve targeting these receptors using monoclonal antibodies, causing a therapeutic blockade (12, 13). Reversing the state of exhaustion in this manner has led to the development of immunotherapies, specifically “checkpoint Inhibitors”, which have been a major breakthrough in cancer treatment until now (14, 15).

Considering the scenario described above, it is conceivable that during the long course of *T. cruzi* infection in humans, T cell exhaustion also takes place. This assumption is supported by a few studies showing dysfunctional T cell responses in other protozoan infections (16, 17), like *Toxoplasma gondii* (18), *Plasmodium* sp (19). and *Leishmania* sp (20, 21). The expression of co-inhibitory receptors during the course of *T. cruzi* infection, their relevance for the elimination of the parasite in the acute phase and their impact on the development of Chagas disease are still controversial. The main reasons are that the acute phase of infection is rarely detected in humans, making it difficult to study big cohorts, and the low number and lack of standardization of experimental mouse models for chronic *T. cruzi* infection (22). *Lasso et al.* have shown, in a cohort of chronic Chagas patients who had been infected for decades, that CD8^+^ T cells undergo successive dysfunction during the development of CD. The authors claim that the progressive hierarchical expression of co-inhibitory receptors on the CD8^+^ T cell compartment and T cell exhaustion are the driving force behind CD (23). The loss of

IFN-γ production by *T. cruzi*-specific CD8^+^ T cells and correlation with the severity of heart disease was also confirmed by other studies (24, 25). The hypothesis of exhaustion of the CD8^+^ T cell response due to the long chronic phase is supported by studies of *T. cruzi*-infected children. These children have significantly shorter infection times and their CD8^+^ T cells show polyfunctional *T. cruzi-*specific responses (26). A series of recent studies in mice highlights disagreements in the field. *Pack et al.* showed no relevant influence of co-inhibitory receptors on CD8^+^ T cells: Despite chronic antigen stimulation, CD8^+^ T cells were able to perform essential functions and contribute to the control of *T. cruzi* parasites in tissue until 100 days post-infection (dpi). The authors concluded that in their model, CD8^+^ T cell exhaustion does not affect the ability of *T. cruzi* to persist in mice (27). On the other hand, *Mateus et al.* showed the opposite in their model of chronically infected mice. They describe monofunctional, antigen-specific CD8^+^ T cells that retain high cytotoxic activity (through the release of Granzyme B (GrB) and perforin) and increased expression of co-inhibitory receptors (mainly CTLA-4 and PD-1) (28). Therefore, whether co-inhibitory receptors on CD8^+^ T cells play a role in keeping the balance between protection and pathology, and whether *in vivo* blocking of inhibitory signaling pathways can improve the clearance of the parasites and prevent the development of CD, has not yet been fully clarified.

Our investigation focused on the establishment of a mouse model with *T. cruzi* strain Brazil to study the acute and chronic phase of infection, and subsequently characterize the co-inhibitory receptor profile of T cells during infection. Furthermore, we performed a longitudinal analysis of specific T cell responses, cytokine production and serum cytokines to gain deeper insights into the course of infection. Of particular interest was to elucidate whether therapeutic intervention, by blocking highly expressed inhibitory pathways like TIM-3 with monoclonal antibodies, has an immunomodulatory effect during infection and whether this can influence the persistence and thus the development of CD. Since a high percentage of TIM-3^+^CD8^+^ T cells were identified, therapeutic intervention was conducted at different time points using monoclonal blocking antibodies against TIM-3 to improve the elimination of parasitic reservoirs. We found that only the long-lasting chronic infection (>2 years) gave rise to dysfunctional CD8^+^ T cells in the tissue of parasite persistence. These CD8^+^ T cells exhibited high expression of the inhibitory receptor PD-1 and co-expressed the transcription factor TOX, which plays a crucial role in the generation of exhausted CD8^+^ T cells and the maintenance of their exhausted phenotype. We suggest that these PD-1^+^ TOX^+^ CD8^+^ muscle infiltrating lymphocytes might ensure an optimized balance during chronic infection.

## Materials and Methods

### Mice

C57BL/6J and RAG1^−/−^ mice were bred at the animal facility of the Bernhard Nocht Institute for Tropical Medicine, Hamburg, Germany. Animals were kept in individually ventilated cages, in a temperature-controlled (22  ±  2°C) and pathogen-free animal facility under biosafety level 3 laboratory conditions (BSL-3). The light/dark cycle was set to a period of 12 hours. Mice received food and water *ad libitum*. Age-matched (7 weeks of age) female mice were used for all experiments.

### Cell Line

86HG39 cells were used to keep parasites alive *in vitro*. Cells were maintained at 37°C 5 % CO_2_ in complete RPMI (cRPMI, PAN Biotech: 10 % fetal calf serum (FCS), 1% L-glutamine and 0.5 % gentamycin (Capricorn Scientific) and were sub-cultured once a week after 5 min treatment with Trypsin-EDTA (Capricorn Scientific).

### Parasites

To recover cryopreserved blood trypomastigotes, the vials were thawed and incubated with a monolayer of 86HG389 cells (29) overnight. Non-infective parasites were removed by collecting cell culture media, the cell monolayer was washed and fresh complete RPMI was added. *T. cruzi*-infected 85HG39 cell culture flasks were maintained at 37°C, 5 % CO_2_ until trypomastigotes were released again. The time until 86HG39 cells burst was 6 days for *T. cruzi* Brazil (30).

### *T. cruzi* Infections

To increase the infectivity of the parasites, cell culture-derived trypomastigotes were passaged once in RAG^−/−^ mice. Subsequently, fresh blood of passage mice was collected by heart puncture at the peak of infection using heparinized syringes. Fresh blood diluted in PBS was used to infect the mice groups used for experiments intraperitoneally (i.p.). The infection dose contained 2 x 10^3^ trypomastigotes in 200 μl PBS per mouse. Not infected controls (n.i.) only received PBS. All mice groups and controls were infected at the same time under BSL-3 conditions. Mice were controlled daily during the experiments. Endpoint analysis took place on the following days post-infection (dpi): 15, 30 (acute stage) and 60, 100, 250 (early chronic stage) and 800 (late chronic stage). For this, mice were anesthetized by CO_2_/O_2_ overexposure and sacrificed by cervical dislocation. Organs (heart, liver, skeletal muscle, and spleen) were harvested. Samples were stored in liquid nitrogen until DNA isolation. Spleen and muscle tissue were isolated for flow cytometry. Blood was collected *via* heart puncture for serum analysis.

### Analysis of Parasitemia in Blood

To determinate free circulating parasites, 2 μl blood was collected from the tail vein, diluted (1:10) in erythrocyte lysis buffer (10 nM tris pH 7.2, 0.15 M ammonium chloride) and incubated for 5 min. Parasites were counted in a 0.02 mm Neubauer cell chamber.

### Quantification of *T. cruzi* Tissue Burden Using Real-Time PCR

Liquid nitrogen-stored tissue samples were thawed and equilibrated to RT. 25 mg were homogenized in 200 µl lysis buffer and proteinase K (Qiagen) in Precellys ceramic Kit tubes and using a Precellys 24 homogenizer (Peqlab, Erlangen, Germany). DNA isolation was performed according to the manufacturer’s specifications using a QIAamp DNA Mini Kit (Qiagen). To assess the purity and quantity of DNA we used a NanoDrop^®^ spectrophotometer (Thermofischer). qPCR reactions were prepared using the QuantiTec SYBR Green PCR Kit (Qiagen) and run on a Rotor-Gene 3000 (Corbett Research, Sydney, Australia). Each reaction was performed in a final volume of 20 µL, containing 50 ng DNA and 0.5 µM each of *T. cruzi* specific primer 121 and 122. Primers target the minicircle variable region from kinetoplast DNA (kDNA) and amplify a 330 bp fragment (31).

121 Fwd: 5´-AAATAATGTACGGGKGAGATGCATGA-3´ and 122 Rev: 5´-GGTTCGATTGGGGTTGGTGTAATATA- 3´. Mouse-specific primers targeting murine GAPDH were used (Fwd: GTCGGTGTGAACGGATTTGG, Rev: TTCCCATTCTCGGCCTTGAC) (32). Thermal profile as previously published (33). The parasite load was calculated automatically using the Rotor-Gene 6000 Series Software 1.7 (Corbett research/Qiagen) by plotting the Ct values against standards of known concentration. GAPDH was used to correct the initial DNA sample mount. qPCR standards were generated by spiking tissue homogenates from n.i. mice with cell-cultured *T. cruzi* trypomastigotes. Afterwards, spiked DNA was serially diluted with DNA isolated from n.i. mice tissue. Standards ranged from 10^7^ to 10^-3^ (limit of detection; LOD) parasite equivalents per 50 ng of total DNA. A standard curve was generated for each organ.

### Preparation of Single-Cell Suspensions From Tissue

Single-cell suspensions of splenic cells were obtained as described in (34). Briefly, spleens were mashed through a 70 μm strainer into cRPMI medium. Erythrocyte lysis was subsequently performed for 5 min at room temperature (RT). Cells were washed with PBS and filtered through a 50 μm strainer. Muscle cells were isolated as described in (35) with some changes. The tissue was dissected from both hindlimbs. Connective and fatty tissue was removed, samples were washed in cold PBS and minced into fine pieces, then digested in 5 ml of digestion medium (0.2% Collagenase type II (2 mg/ml) and 0.05 % Dispase (0.5 mg/ml) in DMEM at 37°C for 40 min. Finally, cells were resuspended in an appropriate volume of PBS and counted.

### Flow Cytometry

Antibodies used for flow cytometric analyses are listed in **(** **Table 1****)**. Single-cell suspensions were first stained with H-2kb-ANYKFTLV Dextramer (Immundex) in PBS + 2 % FCS for 15 min at RT. Subsequently, surface staining antibodies mixed in Fc-block for surface epitopes were added for 30 min at 4°C. For intracellular cytokine staining, cells were fixed and permeabilized with Foxp3/Transcription Factor Buffer Set (ThermoFisher) for 45 min at RT. After fixation and permeabilization, intracellular staining was performed for 60 min at 4°C. Measurements were performed on a BD Fortessa, a BD LSR II (Beckton Dickinson) Cytometer, and a Cytek Aurora. Flow data were analyzed using the FlowJo 10.8.1 software. Gates were set according to fluorescence minus one (FMO) controls. The gating strategy is depicted in the corresponding **Supplementary Figure**.

**Table 1:** List of used antibodies.

Marker	Clon	Dilution	Fluorochrome	Manufacturer
CD3	145-2C11	1:200 1:300 1:300 1:200	AF488 BUV395 PE eFluor 610	Biolegend BD BD Invitrogen
CD3	17A2	1:300	Pe/Cy7	Biolegend
CD4	RM4-5	1:400	V500	Biolegend
CD8	53-6.7	1:200	AF700	Biolegend
CD8	53.67	1:400	V450	eBioscience
CD44	IM7	1:400	PE/Cy7 AF700 BV421	Biolegend
CD45	30.F11	1:300	APC-Cy7	BD
CD45.1	A20	1:200	AF700	Biolegend
CD45.2	103	1:200	APC/Cy-7	Biolegend
CD62L	Mel-14	1:300	PerCP PerCp-Cy5.5 Pe	Biolegend
CD69	H1.2f3	1:100	BV785	Biolegend
CD107a	1D4B	1:200	BV421	Biolegend
LAG-3 (CD223)	C9B7W	1:200	PerCP-Cy5.5	Biolegend
PD-1 (CD279)	RMP1-30	1:200	PE/Cy7	Biolegend
Tim-3 (CD366)	RMT3-23	1:200	APC Pe-Cy7	Biolegend
IFN-γ	XMG1.2	0,3 µL/*well*	AF 488	Biolegend
Granzym B	NGZB	1:200	Pe610	Invitrogen
TNF-α	MP6XT22	1:200	AF700	Biolegend
TOX	REA473	1:100	PE	Miltenyi Biotec
H-2kb- ANYKFTLV Dextramer	---	1:50	PE	Immundex

### *T. cruzi*-Specific *Ex Vivo* Stimulation

Stimulation of *T. cruzi*-specific CD8^+^ T cells was performed with *T. cruzi* protein lysate from a mix of *T. cruzi* trypomastigotes and amastigotes as described in (26) with some modifications. In brief, cell culture-derived trypomastigotes from the Brazil strain were cultured overnight in Dulbecco’s modified Eagle medium (Mediatech; pH 5.0) to transform them into amastigotes. After that, the amastigote and trypomastigote mix was frozen at −196°C and thawed at 56°C four times. After that, the sample was subjected to sonication. The supernatant was collected after centrifugation at 12,000 *x g* and sterile-filtered, then the protein concentration was determined. To stimulate immune cells from the muscle, C57BL/6-Ly5.1 splenocytes were incubated in cRPMI (10 % FCS, 1 % L-Glutamine, 0.5 % Gentamycine) with *T. cruzi* lysate for 5 h. Subsequently, muscle single-cell suspension was added and co-cultured with the antigen-pulsed splenocytes for 5 h at 37°C with 5 % CO_2_. To detect the cytokines IFN-γ, TNF-α and GrB, intracellular staining was performed.

### Cytokine Analysis

Serum samples were obtained by agglutination for 15-20 minutes at RT in Eppendorf tubes followed by centrifugation for 15 min at 5650 *× g* and stored at −20°C. A custom bead-based Immunoassay LEGENDPLEX™ Murine Cytokine Panel (Customized 13-plex from Biolegend) was performed according to the manufacturer’s instructions. Samples were read on a BD Accuri™ C6 Flow Cytometer.

### *In Vivo* Antibody Treatments

TIM-3 neutralization was performed *in vivo* using 100 μg Ultra-LEAF™ (Low Endotoxin, Azide-Free) α-TIM-3 (Biolegend, monoclonal rat IgG) per mouse, injected intra-peritoneally (i.p.) at different time points. Control mice were injected with the corresponding Rat IgG2 a,k (Biolegend).

### Statistical Analysis

Statistical significances were analyzed using Graphpad Prism V9 (Graphpad Software, San Diego, USA). Data were analyzed for normal distribution before analysis. All data are shown as mean or mean ± SEM. The statistical significance was determined using a Kruskal–Wallis test with a posthoc Dunn’s test. Correlation analysis was conducted using a Pearson Correlation test. Statistical assumptions were made at 95% confidence level and *p < 0.05, ** p < 0.01, ***p < 0.001 and ****p < 0.0001. If another test was used, it is indicated in the figure legend.

## Results

### Kinetics of Parasite Load Confirm that *T. cruzi* Persists Concealed in Skeletal Muscle

**Figure 1:**
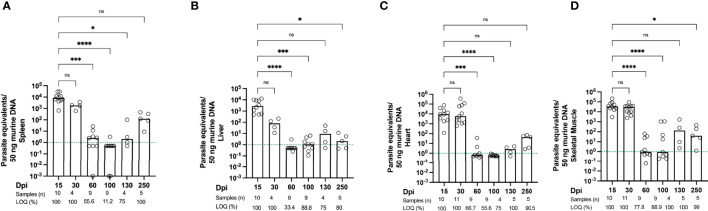
Kinetics of parasite load confirm that *T. cruzi* persists in skeletal muscle. Parasite tissue burden was determined by qPCR. Parasite equivalents per 50 ng DNA were analyzed between 15 dpi (acute phase) and 800 dpi (chronic phase). **(A)** Spleen, **(B)** liver, **(C)** heart and **(D)** sk. muscle (n ≥ 4-23 mice per group). Parasite loads below the limit of quantification (LOQ) were set to LOQ/2, i.e. 0.5 parasite equivalents per 50 ng of DNA. The green dotted line indicates the limit of quantification, which means that samples below this line are still positive, but the parasite load cannot be quantified accurately. 10^-3^ is the limit of detection, as described in Methods. Data were compared using the Kruskal-Wallis test and Dunn’s multiple comparison test. All data are representative of 10 independent experiments. Values are presented as mean. *p < 0.05, ***p < 0.001, ****p < 0.0001, n.s. not significant.

To directly evaluate how reliable our experimental infection was and how successful the parasite control is in our mouse model, we analyzed the parasite burden in different organs: Spleen, liver, heart, and skeletal muscle **(** **Figure 1****).** In line with the results of other groups such as *Lewis et. al*, obtained using visual techniques (33, 36), our infection resulted in high amounts of parasites disseminated throughout the whole organism during acute infection. Initially, during the acute phase, a high parasite load was recorded in all tissues (15-30 dpi). Subsequently, a strong reduction in parasite load was observed over time, with the lowest parasite burden at 60 and 100 dpi, where it could barely be detected in spleen, liver, heart and muscle. However, the parasite burden increased again at later time points. At 800 dpi, several mice displayed a high parasite burden in the muscle. This suggests that although *T. cruzi* can successfully infect all the organs studied, the skeletal muscle is strongly affected and may serve as a niche for the persistence of the parasites **(** **Figure 6A****).**

### Cytokine Expression Profile Reveals a Strong Inflammatory Response Over Time

**Figure 2:**
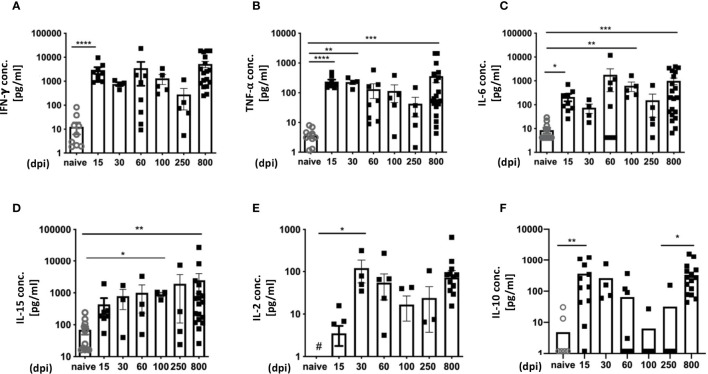
Cytokine expression profiles reveal a strong inflammatory immune response over time. Analysis of cytokines in serum over the course of infection with *T. cruzi* was performed with a bead-based immunoassay to quantify multiple cytokines simultaneously with flow cytometry. The concentrations are given in pg/ml **(A)** for IFN-γ, **(B)** TNF-α, **(C)** IL-6, **(D)** IL-15, **(E)** IL-2 and **(F)** IL-10. A total of n=63 mice were used; n.i. n=13; day 15 n=9, day 30 n=4; day 60 n=8, day 100 n = 4, day 250 = 5 and 800 n=20. Results are presented as mean ± SEM. Data from eight independent experiments were compared using the Kruskal-Wallis test and Dunn’s Multiple Comparison Test. # indicates a value below the detection limit of the assay. *p < 0.05, **p < 0.01, ***p < 0.001, ****p < 0.0001.

In terms of immune activation, our infection model is characterized by two completely different phases: The acute and the chronic phase. Accordingly, it is possible to analyze the dynamic nature of cytokine expression during the course of infection in more detail. To this end, we performed a longitudinal analysis of mouse sera from infected mice **(** **Figure 2****)**. As controls, n.i. mice of the same age were analyzed. It is important to emphasize that there was no age-related increase in cytokine expression, as n.i. mice showed no increase in cytokine levels in the sera with increasing age. The pro-inflammatory cytokines IFN-γ, TNF-α and IL-6 were measured as markers for inflammatory processes. During *T. cruzi* infection, IFN-γ was strongly increased over the entire course of the infection. It strongly increased at 15 dpi, during the acute phase, compared to n.i. controls and remained high. The highest levels of IFN-γ were measured in the late chronic phase, at 800 dpi. **(** **Figure 2A****)**. The analysis of TNF-α showed a very similar pattern. It strongly increased during the acute phase of infection, at 15 dpi and 30 dpi, and remained elevated. Like IFN-γ, TNF-α showed a trend to decrease at 60 dpi, 100 dpi and 250 dpi. But, like IFN-γ, the highest levels of TNF-α were measured at 800 dpi, and the differences to n.i. controls were also highly significant at this time point **(** **Figure 2B****)**. TNF-α is a classical marker of chronic inflammatory processes and is associated with the development of heart disease in Chagas patients (37). IL-6 is released after activation of macrophages, but also by neutrophil granulocytes (38). IL-6 is also released by endothelial cells and somatic cells such as fibroblasts after damage (e.g. tissue destruction). In our model, it was markedly elevated during infection **(** **Figure 2C****).** In the acute phase 15 dpi, it was statistically significantly elevated, but not constantly found at this high level, as the concentration fluctuated between different time points. At 60 dpi a scattering was observed: Some animals showed very high levels, while others showed IL-6 levels similar to n.i. control mice. Thus, the difference to the n.i. group is not statistically significant. IL-15 was also analyzed, since it is a pleiotropic cytokine with a broad spectrum of biological functions that can act in an inflammatory or anti-inflammatory manner, depending on the context (39, 40). Interestingly, IL-15 has been reported to be secreted by muscle cells under homeostatic as well as pathological conditions (41, 42) and little is known about its role in *T. cruzi* infection. Examination of serum IL-15 showed a slight increase at 15 dpi compared to n.i. controls, with a trend towards increased levels as the chronic infection progressed. At 100 dpi, the concentration of IL-15 was significantly higher than in the n.i. controls, and IL-15 was also significantly increased in the late chronic phase **(** **Figure 2D****)**. In addition, IL-2 levels were analyzed due to its importance for T cell function by promoting T cell proliferation and differentiation into effector T cells (T_eff_) as well as ensuring survival and optimal function of T memory and regulatory cells. At 15 dpi, IL-2 was elevated but not significantly different from n.i. controls. It was significantly elevated at 30 dpi, at the end of the acute phase, and remained elevated, though with wide fluctuations, throughout the rest of the chronic infection **(** **Figure 2E****)**. Lastly, IL-10 still plays a controversial role during *T. cruzi* infection; it is an anti-inflammatory cytokine, yet an elevated serum IL- 10 concentration is associated with fibrotic changes and cardiovascular disease (43). Analysis of IL-10 showed a strong and significant increase at 15 dpi compared to n.i. controls **(** **Figure 2F****)**. During the course of the infection, IL-10 levels remained elevated until 30 dpi, but from the onset of the chronic phase (60 dpi), there was a decrease in IL-10. The IL-10 concentration continued to decrease in the further course of the chronic phase. However, at 800 dpi a strong increase was measured. In conclusion, these data show that the levels of pro-inflammatory cytokines in particular remained high throughout the whole course of infection in sera of infected mice, despite a strongly reduced parasite burden in the chronic stage.

### Induction of Co-inhibitory Receptors on Splenic CD8^+^ T Cells From Mice With Acute *T. cruzi* Infection

**Figure 3:**
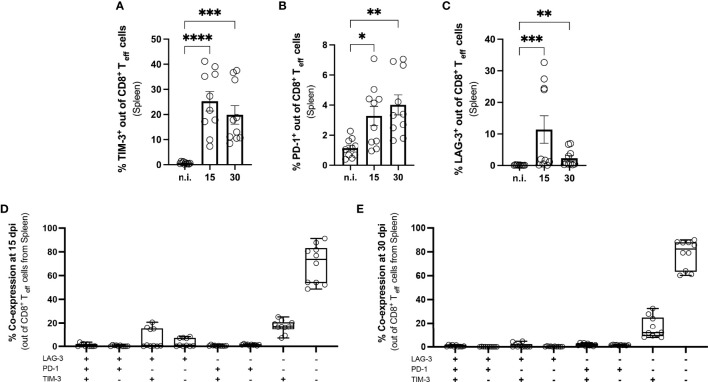
Induction of co-inhibitory receptors on splenic CD8^+^ T cells from mice with acute *T. cruzi* infection. Flow cytometric analysis of splenic CD8^+^ T cells at 15 and 30 dpi. The gating strategy is depicted in S. 1 **(A)** Effector CD8^+^ T cells were defined as CD44^+^ CD62L^-^. Results are presented as percentages of effector CD8^+^ T cells **(A)** for TIM-3, **(B)** for PD-1 and **(C)** for LAG-3. A boolean gating analysis was performed to combine these three markers **(D)** for 15 dpi and **(E)** for 30 dpi. Data are from three independent experiments. Data in **(A–C)** were compared using the Kruskal-Wallis test and Dunn ís multiple comparison test. Results are presented as mean ± SEM (n.i. n = 10; infected day 15 n = 10; day 30 n = 10). *p < 0.05, ** p < 0.01, ***p < 0.001, ****p < 0.0001.

We first determined whether co-inhibitory receptors were induced during experimental *T. cruzi* infection and analyzed the expression of PD-1, LAG-3, and TIM-3 on splenic CD8^+^ T cells at 15 and 30 dpi using flow cytometry. These time points represent the peak of acute infection, with the highest parasitic load in all analyzed tissues. We focused on the activated effector CD8^+^ T cell population, characterized as CD44^+^ CD62L^-^, since polyfunctionality of this subset is necessary to limit further parasite dissemination. The gating strategy and representative dot plots are depicted in **Supplementary Figures 1A–E**. Splenic CD8^+^ T cells showed a significant increase in the expression of all three markers compared to not infected controls (n.i.). Our results revealed a strong and statistically significant increase in the percentage of TIM-3^+^ T cells **(** **Figure 3A****)**. This molecule is an important regulator of T cell-mediated immune responses and its expression correlates with the strength of activation by the T cell receptor. The n.i. controls showed very low expression of this molecule, with on average 0.65 % of CD8^+^ T cells in the spleen being TIM-3^+^. The TIM-3^+^ population decreased towards the end of the acute phase at 30 dpi, but was still statistically significantly higher than in n.i. controls. The frequency of the PD-1^+^ population also increased in comparison to n.i. controls, but this increase was lower than the increase in TIM-3 **(** **Figure 3B****)**. These results are in contrast to earlier findings in other infection models, in which strong T cell activation leads to a high transient PD-1 expression (44). LAG-3 also showed increased expression compared to not infected controls (n.i.), which was significant at 15 dpi and also 30 dpi **(** **Figure 3C****)**. The question arose whether there was co-expression between TIM-3, PD-1 and LAG-3. The proportion of co-expression of these markers within effector CD8^+^ T cells is depicted for 15 dpi in **Figure 3D** and for 30 dpi in **Figure 3E**. In summary, the analysis of CD8^+^ T cells showed that strongly activated CD8^+^ T cells in the spleen transiently expressed the co-inhibitory receptors TIM-3, PD-1 and LAG-3. After the establishment of the early chronic *T. cruzi* infection, when the parasitemia was largely controlled, none of these co-inhibitory receptors could be detected as phenotypic markers for T-cell exhaustion until 250 dpi (**Supplementary Figures 1F–H**). Interestingly, in the late chronic stage at 800 dpi, PD-1^+^ T cells were once again present in spleen and muscle (**Supplementary Figure 1G**).

### High Frequency of TIM-3^+^ CD8^+^ T Cells in the Muscle of Mice With Acute *T. cruzi* Infection

**Figure 4:**
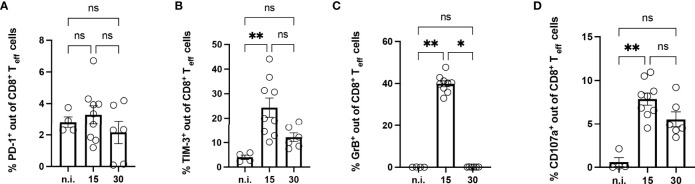
Analysis of CD8^+^ T cells isolated from the muscle of mice with acute *T. cruzi* infection. Phenotypical analysis of CD8^+^ T cells isolated from muscle tissue was performed at 15 and 30 dpi using flow cytometry. Results are presented as percentages of effector CD8^+^ T cells **(A)** for PD-1, **(B)** for TIM-3, **(C)** for GrB, and **(D)** for CD107a. GrB and CD107a were stained *ex vivo* without previous stimulation. GrB was stained intracellularly. Results are presented as mean ± SEM. Data from three independent experiments were analyzed using the Kruskal-Wallis test and Dunn ís multiple comparison tests (n.i. n=4, day 15 n=9, and day 30 n=6). *p < 0.05, **p < 0.01, n.s. not significant.

To investigate the influence of the infection on the CD8^+^ T cells in the muscle, tissue isolated at 15 and 30 dpi was enzymatically digested. The immune infiltrate in the muscle was characterized by flow cytometry (**Figures 4A–D**). In infected mice, the muscle contained a high number of CD3^+^CD45^+^CD8^+^ T cells compared to the muscle of n.i. mice. CD8^+^ T cells in the muscle of infected mice were highly activated and CD44^+^CD62L^-^ **(** **Supplementary Figure 2****)**, similar to the results for the spleen. They also expressed the co-inhibitory receptor TIM-3 to a higher extent. The highest frequency of TIM-3^+^ CD8^+^ T cells was found at 15 dpi and 30 dpi, decreasing at later time points (**Supplementary Figure 1F**). Since only a small percentage of CD8^+^ T cells were PD-1^+^ and the *T. cruzi* infection did not affect the frequency of PD-1^+^ CD8^+^ T cells (**Figure 4A**), we focused on the analysis of TIM-3^+^CD8^+^ T cells (**Figure 4B**). To determine the cytotoxic potential of effector CD8^+^ T cells from muscle tissue, the intracellular expression of GrB and CD107a was determined (**Figures 4C, D**). CD107a, which is exposed on the cell surface as a result of the degranulation of CD8^+^ T cells, serves as a marker for cytotoxicity. While the CD8^+^ T cells of the n.i. mice expressed neither GrB nor CD107a, the frequency of cells expressing these markers was highly increased in infected mice at 15 dpi. It should be emphasized that GrB could no longer be detected at 30 dpi. This loss of GrB production at 30 dpi was also recently described by *Mateus et al.* in spleen cells from *T. cruzi*-infected mice (28). In contrast, analysis of CD107a showed consistently higher levels than the n.i. control without being statistically significant at 30 dpi. It should be noted that the strongest CD107a expression was found in TIM-3^+^CD8^+^ T cells at 15 dpi (not shown). Even if no GrB could be detected, the CD107a expression suggests that degranulation had occurred at 30 dpi.

### Blockade of TIM-3 Did Not Improve Parasitic Clearance in the Muscle

**Figure 5:**
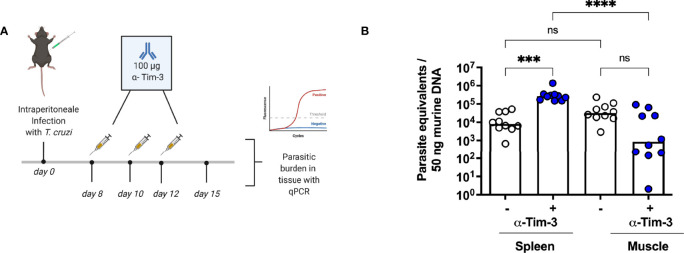
A blockade of TIM-3 increased parasitemia in the spleen but did not improve clearance in the muscle. A blockade using α-TIM-3 was performed. **(A)** Application scheme for the α-TIM-3 treatment. The control group received an isotype antibody using the same regimen. **(B)** The effect of TIM-3 blockade on parasitic load in tissues was analyzed by qPCR. Results are presented as mean. Data from two independent experiments were analyzed using the Kruskal-Wallis test and Dunn ís multiple comparison test (infected mice with α-TIM-3 n = 10, infected mice with isotype antibody n = 10). ***p < 0.001, ****p < 0.0001, n.s. not significant.

Based on the results of the previous experiments, we next aimed to investigate the effect of a therapeutic blockade of TIM-3 using α-TIM-3 during the peak of TIM-3 expression. The expected outcome, as shown in other infection models (45, 46), was an enhanced T cell response, thus more effective elimination of the parasite (47). For this reason, reduction of the parasite load in the tissue was used as a parameter to evaluate the effect. A scheme for the administration of antibodies is shown in (**Figure 5A**). Tissue samples from the spleen, muscle, heart and liver were collected to determine the parasite load. The results of the qPCR for the spleen and muscle are shown (**Figure 5B**). The TIM-3 blockade only resulted in a trend towards a reduction of parasitic burden in muscle tissue, but this difference was not statistically significant compared to the control isotype antibody-treated group. Thus, we could not demonstrate that blocking TIM-3 has an effect on eliminating the parasite in the muscle reservoir. Furthermore, the parasitic load in the spleen was highly increased. Heart and liver tissue did not show differences in the parasitic burden as a consequence of the α-TIM-3 treatment **(data not shown).**

### Loss of Parasite Control in Muscle Tissue in the Late Chronic Phase

**Figure 6:**
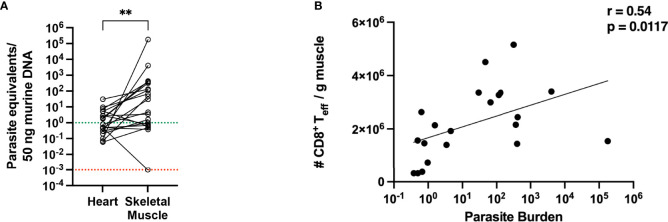
Parasitic burden in muscle tissue is higher than in heart tissue and shows a positive correlation with the number of activated CD8^+^ T cells. **(A)** Parasite equivalents per 50 ng DNA were analyzed from heart and muscle tissues at 800 dpi of infection by qPCR. Data were pooled from two independent experiments (total infected animals n = 20). Results were analyzed for statistical differences with a two-tailored Mann-Whitney test. **(B)** Pearson correlation between parasitic burden in the muscle and the absolute numbers of activated effector CD8^+^ T cells at 800 dpi. **p < 0.01.

Muscle and heart samples were isolated and the parasitic burden was determined by qPCR. In the heart tissue, many samples exhibited a parasite burden below the quantification limit of the qPCR. The mean parasite burden determined for the heart was 3.5 parasites/50 ng heart tissue, while in muscle 8800 parasites/50 ng muscle tissue were found. Comparing the parasite burden in the heart and muscle tissue for each mouse **(** **Figure 6A****)**, the parasite burden is significantly higher in muscle tissue than heart tissue, and there is no correlation between the number of parasites in muscle and the number of parasites in the heart of the same mouse. Furthermore, a large scattering of the values can be seen, ranging from 1 to 1x10^6^ parasites/50 ng DNA in muscle tissue. This number of parasite equivalents per nanogram tissue corresponded to the observed tissue parasite burden in the acute phase **(** **Figure 1****)**. This strongly suggests that, although all mice were in good health, some mice in the infection group were less able to control tissue parasite burden in the skeletal muscle over a longer period. The parasite load and the number of activated CD8^+^ T cells present in the muscle were correlated and the r-value of 0.54 indicates a moderate positive correlation between the parasite burden and the number of activated CD8^+^ T cells in the muscle **(** **Figure 6B****)**.

### Prolonged Infection Leads to Expression of PD-1 and TOX of CD8^+^ T Cells in the Muscle

**Figure 7:**
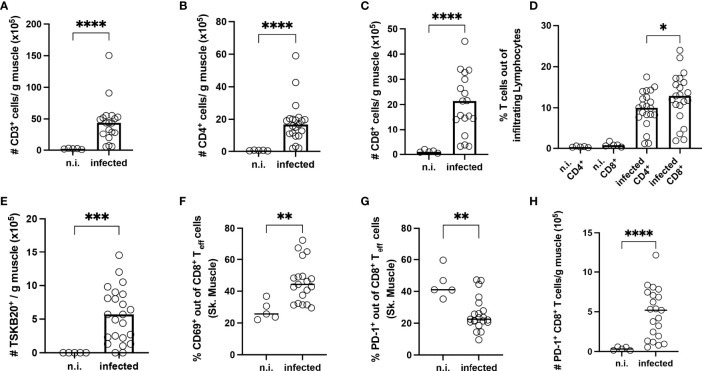
Long-term infection with *T. cruzi* leads to strong infiltration of T cells into the skeletal muscle tissue. T cells were isolated from the skeletal muscle of infected mice at 800 dpi and analyzed by flow cytometry. Upper line **(A–C)** Results expressed as absolute numbers per gram of muscle tissue. **(A)** CD3^+^, **(B)** CD4^+^ and **(C)** CD8^+^ T cells. **(D)** Frequency analysis of infiltrating T cells. Bottom line (E-H): In absolute numbers **(E)** TSKB20^+^ T cells. **(F, G)** Results expressed as a percentage of effector CD8^+^ T cells (T_eff_). T_eff_ population was defined as CD44^+^CD62L. A representative plot of the staining and gating strategy is depicted in S. 3 A-B. In **(F)** CD69, and in **(G)** PD-1. Absolute numbers of PD-1 cells **(H)**. Data are from two independent experiments. Results are presented as mean (n.i. n = 5; infected n = 20) and analyzed by the Kruskal-Wallis test and then by Dunn’s multiple comparison test. *p < 0.05, **p < 0.01, ***p < 0.001, ****p < 0.0001.

Next, the T cells from the muscle were further examined by flow cytometry. Representative plots and the gating strategy are depicted in **Supplementary Figures 3A, B**. In infected mice, a higher infiltration of CD3^+^ T cells could be observed **(** **Figure 7A****)**. The biggest fraction of infiltrating cells was CD8^+^ T cells **(** **Figures 7B–D****)**. Using MHC-I dextramer staining against the immunodominant TSKB20^+^ epitope of the *T. cruzi trans*-sialidase, we also found a strong enrichment of these cells **(** **Figure 7E****)**. CD44^+^CD62L^-^ effector CD8^+^ T cells were examined for the expression of activation markers and co-inhibitory receptors. The frequency of effector cells was significatively increased in infected mice **(** **Supplementary Figure 4A****)**. At this late timepoint, neither TIM-3, LAG-3 or GrB could be detected on CD8^+^ T cells from the muscle of infected mice anymore **(** **Supplementary Figures 4B–D****)**. CD69 is a marker for early T cell activation, but it also marks non-circulating tissue-resident cells in non-lymphoid tissues (48–50). The percentage of CD69^+^ T cells was significantly higher than in n.i. controls **(** **Figure 7F****)**. Additionally, comparing T_eff_ cells from the same mice between the tissues, although the absolute numbers of CD8^+^ T cells in the spleen were comparable to the numbers found in the muscle **(** **Supplementary Figure 4E****)**, the frequency of CD69^+^CD8^+^ T cells was significantly increased only in T_eff_ cells from muscle tissue, whereas T_eff_ cells from the spleen expressed much less CD69 **(** **Supplementary Figure 4F****)**. In addition, there is a moderate negative correlation between the absolute number of CD8^+^ T cells between the spleen and the muscle, suggesting an accumulation of CD8^+^ T cells in the muscle over time **(** **Supplementary Figure 4G****)**. While analyzing the PD-1 expression on the isolated CD8^+^ T cells, it became apparent that a high percentage of the very few CD8^+^ T cells found in the muscle of n.i. mice already expressed PD-1 **(** **Figure 7G****).** However, upon infection, the absolute numbers of CD8^+^ T cells strongly increased **(** **Figure 7H****)**. Thus, upon infection, an increased absolute number of PD-1^+^CD8^+^ T cells was found in the muscle.

In conclusion, a strong accumulation of PD-1^+^CD8^+^ T_eff_ in the muscle was only found after a very long infection period of two years. The high frequency of CD69^+^ CD8^+^ T cells within the muscle, compared to the very low frequency of these cells in the spleen, presents an argument against CD69 induction only as a consequence of an ongoing T cell activation, and supports a tissue-resident phenotype in the muscle. Strikingly, CD8^+^ T cells in the muscle directed against the immunodominant TSKB20 epitope derived from the *trans*-sialidase of *T. cruzi* remained PD-1^-^ (**Supplementary Figure 5A**).

### Effector PD-1^+^ CD8^+^ T Cells Co-Express the Transcription Factor TOX and Show a Reduced Capacity to Produce Cytokines After *Ex Vivo* Stimulation

**Figure 8:**
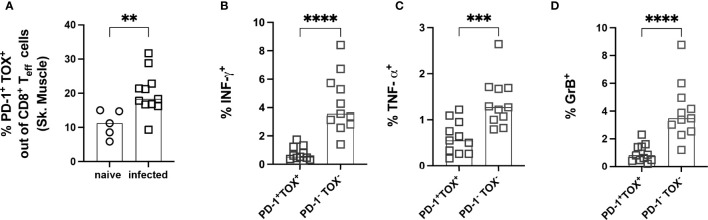
Effector PD-1^+^ CD8^+^ T cells co-express the transcription factor TOX and show a reduced capacity to produce cytokines after *ex vivo* stimulation. Functional analysis of PD-1^+^ TOX^+^ CD8^+^ T_eff_ from muscle tissue. Representative staining for the co-expression of PD-1 and TOX on CD8^+^ T_eff_ cells is depicted in **Supplementary Figure 5B**. **(A)** Co-expression of PD-1 and TOX was quantified and expressed as the frequency of CD8^+^ T_eff_ cells. CD8^+^ T_eff_ cells were stimulated with *T. cruzi* lysate to investigate functionality. IFN-γ **(B)**, TNF-α **(C)** and GrB **(D)** were stained intracellularly. Data are from one experiment and were compared using the Kruskal-Wallis test and Dunn’s Multiple Comparison Test. The results are presented as mean (n.i. = 5, infected n= 11). **p < 0.01, ***p < 0.001, ****p < 0.0001.

To further explore whether the PD-1^+^ T cells were exhausted, the expression of the transcription factor TOX was analyzed. The frequency of PD-1^+^ TOX^+^ CD8^+^ T_eff_ was significantly increased in infected mice **(** **Figure 8A****)**. A representative staining of PD-1 and TOX gated on CD8^+^ T_eff_ cells showing clear co-expression is depicted in **Supplementary Figure 5B**. To test the capacity of PD-1^+^ CD8^+^ T_eff_ cells to produce cytokines, these cells were stimulated *ex vivo*. The gating strategy and representative dot plots are depicted in **Supplementary Figure 6**. Lymphocytes from the muscle of infected mice were co-cultivated with spleen cells isolated from n.i. Thy1.1-congenic C57BL/6 mice. These cells were pre-incubated with *T. cruzi* lysate, since cells from the spleen contain an appropriate number of antigen-presenting cells (APC) to better stimulate the T cells isolated from the muscle. This experimental set-up takes advantage of the fact that the cells from Thy1.1 C57BL/6 mice express the congenic marker CD45.1 **(** **Supplementary Figure 6B****)**, whereas cells from a wild-type C57BL/6 mouse express the CD45.2 allele **(** **Supplementary Figure 6C****)**. Thus, n.i. splenic cells used as APC were CD45.1^+^ and could be discriminated from CD45.2^+^CD8^+^ T cells from muscle tissue of infected mice. The ability of PD-1^+^ TOX^+^ T cells to produce pro-inflammatory cytokines and cytotoxic granules after stimulation was examined. The gating strategy and representative dot plots for this staining are depicted in **Supplementary Figure 6D**. Production of IFN-γ, TNF-α and GrB was found to be impaired **(** **Figures 8B–D****).**

In summary, the experiments in the late chronic phase showed that a very long infection period can indeed lead to an accumulation of dysfunctional CD8^+^ T cells, which are characterized by a high degree of co-expression of the co-inhibitory molecule PD-1 and the transcription factor TOX. TOX, therefore, appears to play a crucial role in generating and maintaining the exhausted phenotype of CD8^+^ T cells in this model. Nevertheless, the CD44^+^CD62L^-^CD69^+^PD-1^+^TOX^+^ T cells infiltrating the muscle still appear to exert control over the parasite burden in the tissue. However, whether this represents an optimal equilibrium that occurs in the course of chronic infection or represents the beginning of a complete loss of pathogen control must be clarified in further studies.

## Discussion

The intracellular protozoan parasite *T. cruzi* is a complex, ancient eukaryotic organism and a versatile pathogen that can persist in the host organism for life. The *T. cruzi* host-parasite interaction is immunologically highly interesting, as the control of the infection is mainly ensured by CD8^+^ T cells. In models of chronic viral infections, it has already been shown that T cells undergo phenotypic changes characterized by a progressive increase and co-expression of co-inhibitory receptors, leading to exhaustion and dysfunctionality. This means that important T cell functions such as cytotoxicity (release of granules and GrB), proliferation and the production of cytokines such as IFN-γ, TNF-α and IL-2 are diminished or even lost (51). Although there is no definitive definition and marker combination to describe exhausted T cells, co-expression of PD-1, LAG-3 and TIM-3 has been extensively described (52, 53). To prevent permanent damage to the host due to the immune response, CD8^+^ T cells must be negatively regulated in their function to ensure immune homeostasis (54). This study aimed to characterize the co-inhibitory molecule profile of CD8^+^ T cells during infection with T. *cruzi* in detail, and to test whether the co-expression of multiple co-inhibitory receptors on CD8^+^ T cells, as a physiological mechanism to avoid immunopathology, favors parasite persistence and disease. To this end, a model was established based on the intra-peritoneal infection of female C57BL/6 mice with T. *cruzi* trypomastigotes of the strain Brazil (DTU I) (55, 56). The acute infection with *T. cruzi* Brazil was characterized by a ubiquitously high parasite load while remaining asymptomatic, as found in humans. This means that these mice did not show severe weight change or signs of acute disease. The parasitemia was successfully controlled within 30 days and led to a highly restricted distribution of parasites in the muscle tissue as a reservoir during the chronic phase. For a long time, tissue preferences leading to a heterogeneous load in different tissue types was described as a characteristic of many *T. cruzi* strains. For example, some strains have been described as myotropic and others as reticulotropic (57). In 2014, the development of highly sensitive bioluminescence imaging techniques by *Lewis et al.* demonstrated that, regardless of the discrete typing units (DTU) affiliation of the *T. cruzi* strain, persistence is mainly restricted to skeletal muscle (33, 58). Our results reproduce the infection courses of other research groups (27, 59). The late chronic infection at 800 dpi was characterized by loss of parasite control in some mice. Taken together, the mouse model established for this study reliably reproduces the infection dynamics described with other techniques and is suitable to investigate the mechanisms leading to persistent infection, which is the most important question in CD research. In this study, it was clearly shown that CD8^+^ T cells from the spleen and muscle are highly effective during the acute phase of infection and are not characterized by the expression of multiple co-inhibitory receptors. First, the results confirm that the T cell response in the infected mice is effective enough to ensure survival and that the CD8^+^ T cell response is concentrated on the immunodominant epitope TSKB20 of the *trans*-sialidase (60). In addition, we found an expression of individual co-inhibitory receptors such as TIM-3 and to a lesser extent of PD-1 and LAG-3 on CD8^+^ T cells. The transient expression of PD-1 on CD8^+^ T cells may also be interpreted as a consequence of their activation (44). LAG-3 was particularly strongly induced at 15 dpi. LAG-3 expression might indicate the involvement of type 1 regulatory T cells (Tr1) (61). These cells have been characterized by LAG-3 and CD49b expression and confer suppressive capacity. However, we could not find T cells with the aforementioned phenotype at any time point during infection. However, it should be noted, that we cannot exclude that CD8^+^ T cells expressing LAG-3 and TIM-3, as found in our study, could have also a suppressive capacity as recently described by *Brandi et al.* using an acute mouse model of malaria (62). CD8^+^ T cells with a similar co-inhibitory-rich phenotype could also be found in patients suffering from acute malaria further highlighting their relevance in acute infections. In our model, TIM-3 was the co-inhibitory molecule that was most strongly induced on CD8^+^ T cells upon infection. TIM-3 was first described on differentiated IFN-γ producing CD4^+^ and CD8^+^ T cells, and induced T cell apoptosis by binding to galectin-9 (63, 64). TIM-3 has also been shown to mark the most exhausted and dysfunctional T cells that arise in chronic viral infections such as HIV or in cancer (9, 65). However, TIM-3 expression, in an acute state, can also be the result of a strong activation (66–68). *Avery et al.* have shown that TIM-3 partially exerts a costimulatory function by amplifying signals in the immunological synapse. It is thought that excessive activation by the TCR and an excessive effector CD8^+^ T cell response disrupt the formation of T cell memory, and that this is how TIM-3 contributes to T cell exhaustion (69). Although blockade of TIM-3 has been shown to enhance the T cell response and restore its functionality in other models (12, 70, 71), in this study the blockade of this molecule in the acute phase of *T. cruzi* infection only resulted in a non-significant change in parasite load in the muscle. The fact that the TIM-3 blockade led to an increase in tissue burden in the spleen may be due to the effects that the TIM-3 blockade exerts on cells of the innate immune system. TIM-3 is expressed on many cells of the innate and adaptive immune system, particularly on macrophages, for which TIM-3 blockade has been shown to increase their number and activation (72). For this reason, it is conceivable that the TIM-3 blockade would allow highly activated macrophages, which have already eliminated many parasites, to accumulate in the spleen. Thus, detection of tissue load by qPCR after blockade could indicate a higher load even though these parasites are no longer vital. Further experiments are needed to verify this hypothesis, since assessing the effects of TIM-3 blockade on other immune cells was not within the scope of this work. The decrease in PD-1 and TIM-3 at the end of the acute phase (30 dpi) can be interpreted as a sign of a resolution of the T cell response. During the transition from the acute to the chronic phase, the amount of antigen decreases sharply, as the chronic infection is associated with very low parasitemia. The results of our study cannot support the hypothesis that complete elimination of *T. cruzi* is prevented due to T cell exhaustion or even an over-regulated T cell response, since CD8^+^ T cells were not inhibited in their effector function during the acute phase of infection, as indicated by the successful control of parasite proliferation. Thus, the persistence of *T. cruzi* in infected mice is not the result of excessive negative regulation or dysfunctional CD8^+^ T cells. The data shown here provide no evidence for a limitation of CD8^+^ T cell function due to co-inhibitory receptors expressed during the acute phase of *T. cruzi* infection. The study of the early chronic phase showed that CD8^+^ T cells are crucial for the long-term control of *T. cruzi* infection. CD8^+^ T cells showed no expression of co-inhibitory receptors in chronically infected mice. Studying CD8^+^ T cells in the muscle, being the tissue of persistence, is highly relevant, as it is the site where antigen exposure is highest for an extended period of time, which could contribute significantly to T cell exhaustion. During long-term infection, a population of activated effector CD44^+^CD62L^-^ CD8^+^ T cells massively infiltrate the muscle and may continuously control the spread of the parasites. This study shows, for the first time, that a proportion of these effector CD8^+^ T cells in the muscle co-express PD-1 and the transcription factor TOX. TOX is a recently described transcription factor that epigenetically determines T cell exhaustion (73, 74). In line with this, after antigen-specific restimulation, effector PD-1^+^TOX^+^CD8^+^ T cells exhibited a marked loss of effector functions, producing significantly less IFN-γ, TNF-α and GrB compared to their PD-1^-^TOX^-^ counterparts. Thus, the emergence of these T cells with an exhausted phenotype is the result of parasite persistence rather than its cause. This hypothesis is strongly supported by recent studies showing that an increase in MHC-I expression in mouse muscle leads to an enhanced CD8^+^ T cell-mediated reduction in parasite burden. However, prolonged overexpression of MHC-I resulted in a loss of parasite control and a massive increase in exhausted PD-1^+^CD8^+^ T cells (75). It is noteworthy that CD8^+^ T cells recognizing an immunodominant epitope of the *T. cruzi trans*-sialidase were also found in the muscle, but, in contrast to other CD8^+^ T_eff_, they did not express PD-1 at all. The reason for this dichotomy remained unclear, but it is tempting to speculate that the lack of PD-1 expression might contribute to their immunodominance. However, it has already been shown that these *trans*-sialidase-specific CD8^+^ T cells did not contribute to protection, despite their high number (76, 77). The data shown here prove that a very long chronic infection with *T. cruzi*, even in the mouse model, can lead to a loss of function of CD8^+^ T cells. Moreover, although these T cells express only one of the co-inhibitory receptors analyzed here, namely PD-1, the co-expression of TOX marks them as exhausted cells. Alternatively, they may have adapted to the situation of chronic infection and only retained the necessary functionality to control the pathogen without harming the host (54).

During the course of the entire infection, there was a strong systemic inflammation, which was evident from the examination of cytokines in the serum, even though the chronic infection established in this mouse model followed an asymptomatic course. Modeling Chagas disease using mice remains a challenge, but it is nevertheless essential to identify factors leading to pathology. It remains unclear whether the very long chronic courses of infection seen in humans (30 years), leading to a deterioration of the immune response, can be reproduced during the shorter lifespan of mice (78). One limitation of this study is that we did not address sex-specific differences, although sex might influence the course of infection or the development of heart disease. There is no clear evidence of sex-specific differences in Chagas disease. However, it has been shown that there are increased pathological changes, including myocardial damage in male mice (79) and that female mice are more resistant to *T. cruzi* infection than male mice (80). In addition to sex differences, studies have shown a high prevalence of comorbidities in Chagas patients, probably due to advanced age (81). The infection with *T. cruzi* triggered a strong systemic induction of pro-inflammatory cytokines. IFN-γ, which remained elevated throughout the course of the infection, plays a central role in combating *T. cruzi*. In IFN-γ deficient mice, a *T. cruzi* infection, even with a low-virulence strain, is lethal (82). Increased IFN-γ polarizes the immune response towards differentiation into T_H_1 T cells. It plays a major role in the defense against intracellular pathogens by activating macrophages and differentiating cytotoxic CD8^+^ T cells, which are essential for the elimination of *T. cruzi*. Macrophages increase antigen presentation, produce nitric oxide and reactive oxygen radicals as well as nitrogen-dependent microbicidal defense systems. Finally, macrophages activated by IFN-γ produce more TNF-α. A robust immune response by IFN-γ-producing CD4^+^ T cells is necessary to control parasite replication, and loss of this cytokine also leads to a loss of these protective mechanisms. This has been shown in studies of Chagas patients who have an HIV co-infection or are immunosuppressed (83, 84). The pathological effects of chronically elevated IFN-γ levels, as found in infected muscle tissues, are also still unclear. IFN-γ-induced cytokine-mediated inflammation has been observed in Chagas patients and has been connected to cardiac pathology (85, 86). TNF-α is mainly secreted by activated monocytes and macrophages, and also by T_H_1 helper cells and CD8^+^ cytotoxic T cells in small amounts. It leads to strong endothelial activation by inducing adhesion molecules and intensifying local inflammation. In many chronic inflammatory states, TNF-α induces cachexia, i.e., loss of muscle mass through catabolic processes. Patients with high levels of IFN-γ and TNF-α were found to have a higher risk of disease progression (87, 88). The most interesting cytokine in the context of *T. cruzi* infection in this study was IL-15, which is also strongly induced by the infection and remains at a very high level throughout the course of the infection. The source of the large amounts of IL-15 remains unclear. IL-15 fulfills several functions, such as supporting the survival of T cells and the activation of NK cells, both of which are essential cell types for a robust immune response against *T. cruzi* (40, 89). A recent study by *Wu et al.* has shown that CD8^+^ T cells are functionally impaired or depleted in chronic viral infections and that this phenomenon is accompanied by an unwanted loss of muscle mass. The production of IL-15 by muscle tissue has already been described (41), however, the authors show that the production of muscle IL-15 regulates T cell exhaustion. IL-15 produced by muscle tissue supports the recruitment of CD8^+^ T cells within the muscle microenvironment, and this protects them from overwhelming activation during inflammation. *Wu et al.* showed that the CD8^+^ T cells in muscle have a higher proliferative potential than T cells in other secondary lymphoid organs, such as the spleen. When required, T cells from the muscle re-enter the lymphoid tissue and differentiate into functional effector T cells. The authors postulate that muscle-specific IL-15 production counteracts T cell exhaustion by protecting the proliferative potential of T cells from inflammation, and by protecting the T cell compartment in lymphoid organs (42). In our model of *T. cruzi* infection, T cell exhaustion occurs very late. At early time points CD8^+^ T cells are fully functional and control parasite replication. However, residual parasite burden in specific niches like the muscle were not cleared leading to a continuous presence of antigen. It remained to be studied based on the results of this work, if the muscle is the source of the elevated IL-15 levels in the serum which might delay exhaustion of CD8^+^ T cells despite chronic antigen exposure and if this also counteracts atrophy of the muscle induced by TNF-α as described by O´Leary et al. (90).

Overall, the model established in this study is excellent to investigate parasite persistence mechanisms in the muscle, whereas the pathological processes in the heart do not yet correspond to those in humans. The persistence does not seem to be the result of CD8^+^ T cells dysfunction, which can control parasitemia over a long period very efficiently. However, a few parasites in the muscle evade this control for as yet unexplained reasons. Deciphering these mechanisms holds the potential for new immunotherapies to be developed in order to further reduce the number of persistent parasites in the muscle, or even eliminate them completely.

## Data Availability Statement

The raw data supporting the conclusions of this article will be made available by the authors, without undue reservation.

## Ethics Statement

The animal study was reviewed and approved by Federal Authority of the State of Hamburg (Hamburger Behörde für Justiz und Verbraucherschutz, Antrag 52/17).

## Author Contributions

TJ conceived and supervised the study; RG conceived and conducted all mouse experiments, collected and analyzed data and wrote the manuscript. Both authors revised the manuscript and approved the submitted version.

## Funding

RIG was supported by a merit-based scholarship from the Konrad Adenauer Foundation, Germany (Promotionsförderung der Konrad-Adenauer-Stiftung) during her PhD.

## Conflict of Interest

The authors declare that the research was conducted in the absence of any commercial or financial relationships that could be construed as a potential conflict of interest.

## Publisher’s Note

All claims expressed in this article are solely those of the authors and do not necessarily represent those of their affiliated organizations, or those of the publisher, the editors and the reviewers. Any product that may be evaluated in this article, or claim that may be made by its manufacturer, is not guaranteed or endorsed by the publisher.

## Supplementary Material

Supplementary Figure 1 Representative gating strategy for T cell analysis (spleen) acute phase and longitudinal analysis. **(A)** Representative strategy to define effector CD3^+^CD8^+^ T cells based on the expression of CD44 and CD62L. Co-inhibitory receptors were gated within CD44^+^CD62L^-^ CD8^+^ T cell population (highlighted in blue). Representative gating strategy of **(B)** TIM-3, **(C)** PD-1, **(D)** LAG-3 and **(E)** TSKB20 induced by the infection with *T. cruzi* 15 and 30 dpi. Longitudinal analysis of co-inhibitory receptors in CD44^+^CD62L^-^ CD8^+^ T cells from spleen and sk. muscle for **(F)** TIM-3, **(G)** PD-1 and **(H)** LAG-3.

Supplementary Figure 2 Representative gating strategy for T cell analysis in the muscle in the acute phase. Representative strategy to define effector CD3^+^ CD8^+^ T cells based on CD44^+^ and CD62L^-^expression. Co-inhibitory receptors were gated within CD44^+^CD62L^-^ CD8^+^ T cell population. Representative gating strategy of **(B)** TIM-3, **(C)** GrB and **(D)** CD107a induced by the infection with *T. cruzi* at 15 and 30 dpi. Absolute numbers of infiltrating lymphocytes in muscle per gram tissue at 15 dpi **(E)** CD3^+^, **(F)** CD8^+^, **(G)** TSKB20^+^. **(H)** Percentage of TIM-3^+^ and PD-1^+^ out of TSKB20^+^ effector CD8^+^ T cells from muscle at 15 dpi. **(I)** Boolean gating analysis of TIM-3, GrB and CD107a. The box plots show the median and range of each combination (15 dpi is depicted in black and 30 dpi in blue). Data from two experiments were compared using the Kruskal-Wallis test and Dunn’s Multiple Comparison Test.

Supplementary Figure 3 Representative gating strategy for T cell analysis in muscle during the chronic phase. Representative strategy to define effector CD3^+^ CD8^+^ T cells based on CD44 and CD62L expression. Co-inhibitory receptors were gated within CD44^+^CD62L^-^ CD8^+^ T cell population (highlighted in blue). Representative gating strategy of **(A)** not infected and **(B)** *T. cruzi-*infected mice 800dpi.

Supplementary Figure 4 Analysis of co-inhibitory receptors on effector CD8^+^ T cells from muscle tissue. Upper line: Flow cytometric analysis of T_eff_ from muscle 800 dpi. Results are shown as percentages. **(A)** T_eff_ cells out of CD8^+^ T cells, **(B)** TIM-3, **(C)** LAG-3 and **(D)** GrB gated within CD8^+^ T_eff_. Bottom line: T cells were isolated from the spleen of infected mice at 800 dpi and analyzed by flow cytometry. Results are expressed as absolute numbers per organ. **(E)** CD8^+^ T cells. **(F)** Comparison of percentage of CD69^+^ cells in CD8^+^ T_eff_ cell population from spleen and muscle of infected mice. **(G)** Pearson correlation between absolute numbers of CD8^+^ T_eff_ cells from muscle per gram and absolute CD8^+^ T_eff_ cells from spleen (organ). Data from two independent experiments are presented as mean. Results were analyzed for statistical differences with a two-tailored Mann-Whitney test (n.i. n=5; Infected n=20).

Supplementary Figure 5 Differential PD-1 expression between immunodominant *trans*-sialidase-specific and bulk *T. cruzi*-specific CD8^+^ T cells. CD8^+^ T_eff_ from muscle tissue of *T. cruzi*-infected mice 800 dpi were stained using an MHC-I dextramer against the immunodominant TSKB20 epitope and co-stained with anti-PD-1. **(A)** Bulk CD44^+^CD62L^-^CD8^+^ T_eff_ and *T. cruzi*-specific TSKB20^+^ CD8^+^ T_eff_ from the muscle of *T. cruzi*-infected mice at 800 dpi were surface stained with anti-PD-1. **(B)** TOX was intracellularly stained in bulk CD44^+^CD62L^-^CD8^+^ T _eff_ cells isolated from the muscle of *T. cruzi* infected mice and is positively correlated with the expression of PD-1.

Supplementary Figure 6 Stimulation of T cells derived from muscle tissue of infected CD45.2 mice with *T. cruzi* lysate-pulsed splenocytes from CD45.1 mice as APC. **(A)** Gating strategy of not infected splenocytes from CD45.1 mice used as antigen-presenting cells (highlighted in orange). **(B)** PD-1 and TOX gating on infected cells in CD45.1^+^ cells and their capability to produce IFN-γ. **(C)** Gating strategy for infected muscle cells from CD45.2 mice, defined as “effector cells” (highlighted in blue). **(D)** Representative plot of the PD-1 and TOX co-expression gated on CD44^+^CD8^+^ T cells. **(E)** Comparison of the capability to produce IFN-γ, TNF-α and GrB between PD-1^+^TOX^+^ and PD-1^-^TOX^-^ T cells. **(F)** FMO controls were used to set the gates for CD44, PD-1 and TOX.
