# Supplementary material for: Exhausted PD-1+ TOX+ CD8+ T Cells Arise Only in Long-Term Experimental Trypanosoma cruzi Infection
Source: Front Immunol. 2022 Jun 3;13:866179. doi: 10.3389/fimmu.2022.866179 (PMC9203896; doi:10.3389/fimmu.2022.866179)
Supplement: Supplementary Figure 1 — Representative gating strategy for T cell analysis (spleen) acute phase and longitudinal analysis. (A) Representative strategy to define effector CD3+CD8+ T cells based on the expression of CD44 and CD62L. Co-inhibitory receptors were gated within CD44+CD62L- CD8+ T cell population (highlighted in blue). Representative gating strategy of (B) TIM-3, (C) PD-1, (D) LAG-3 and (E) TSKB20 induced by the infection with T. cruzi 15 and 30 dpi. Longitudinal analysis of co-inhibitory receptors in CD44+CD62L- CD8+ T cells from spleen and sk. muscle for (F) TIM-3, (G) PD-1 and (H) LAG-3. [file DataSheet_1.pdf]

Figures and Materials to

Exhausted PD-1^{+} TOX^{+} CD8^{+} T cells arise only in long-lasting experimental *Trypanosoma cruzi* infection

Rosa Isela Gálvez & Thomas Jacobs*

*Correspondence to: Tjacobs@bnitm.de (T.J.)

Supplementary Materials S1 – S6
Table T1

S1. Gating Strategy and exemplary dot blots

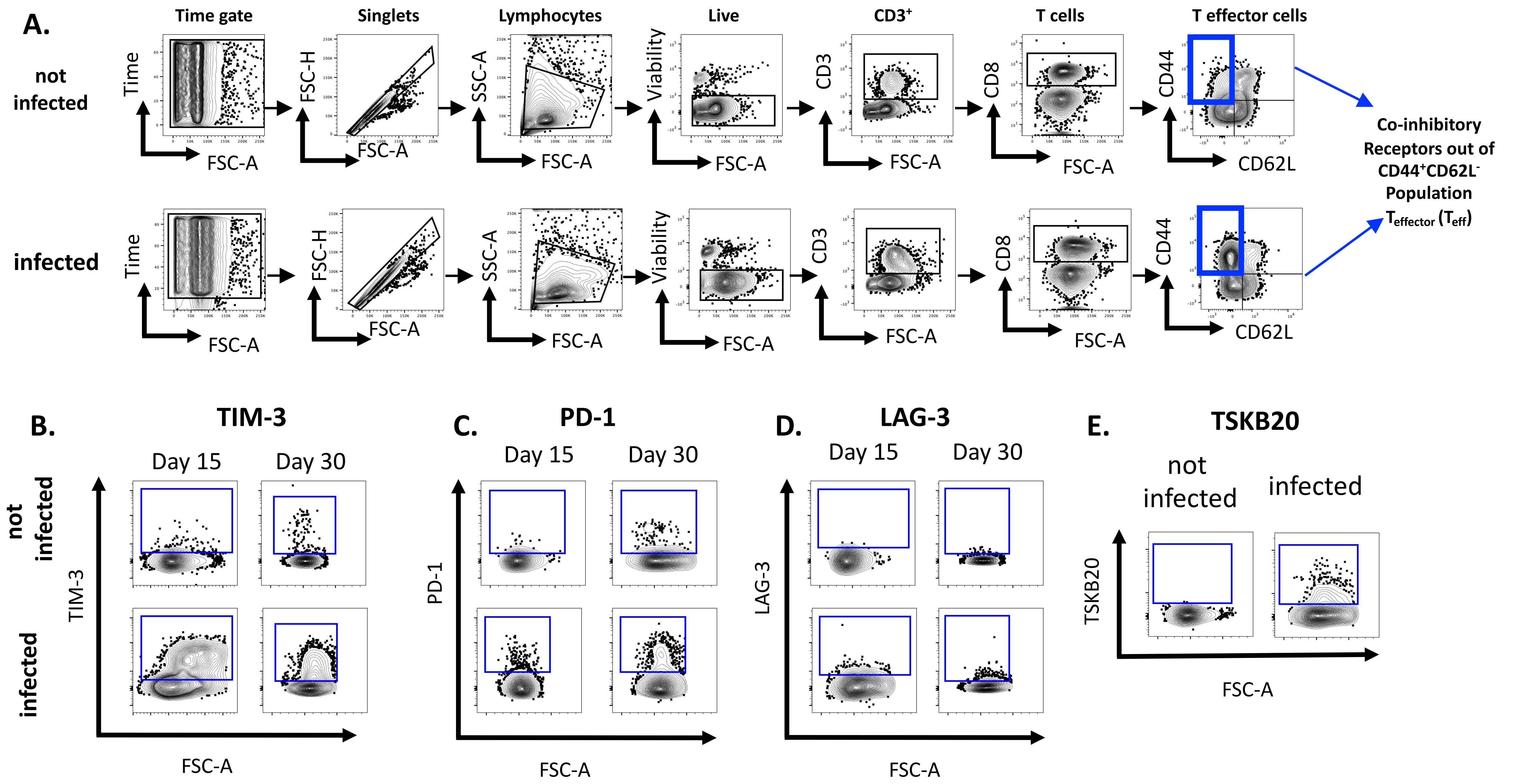

TIM-3

F.

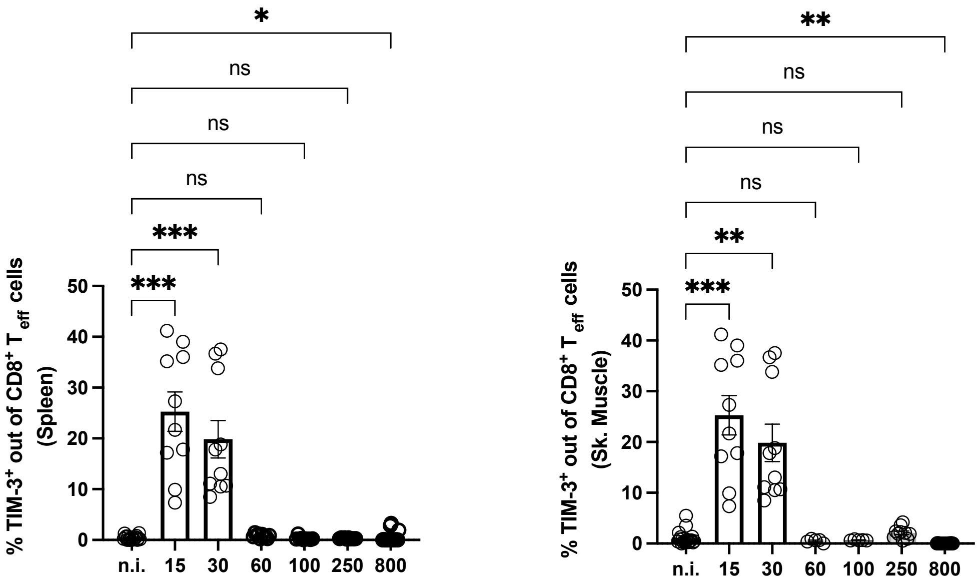

PD-1

G.

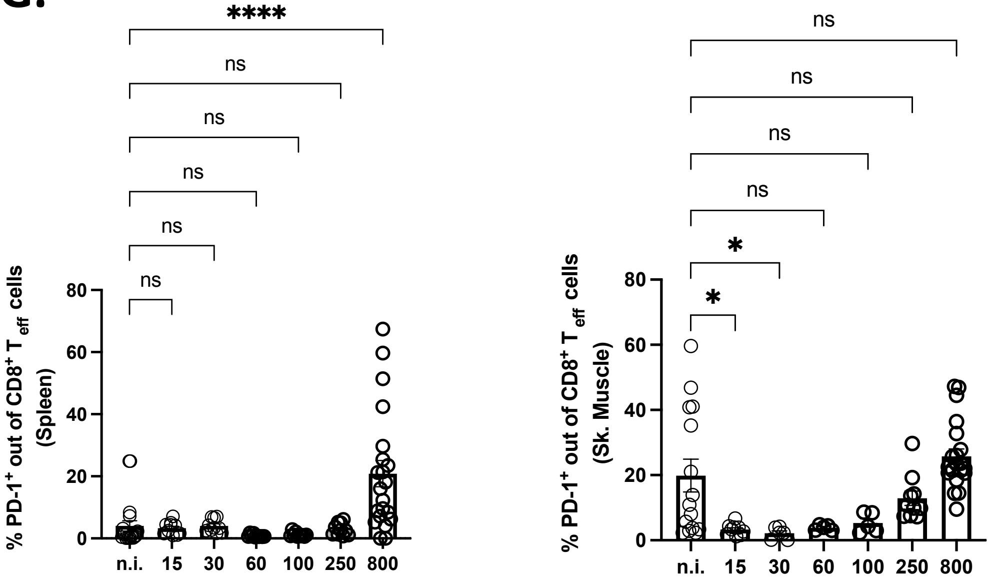

H.

LAG-3

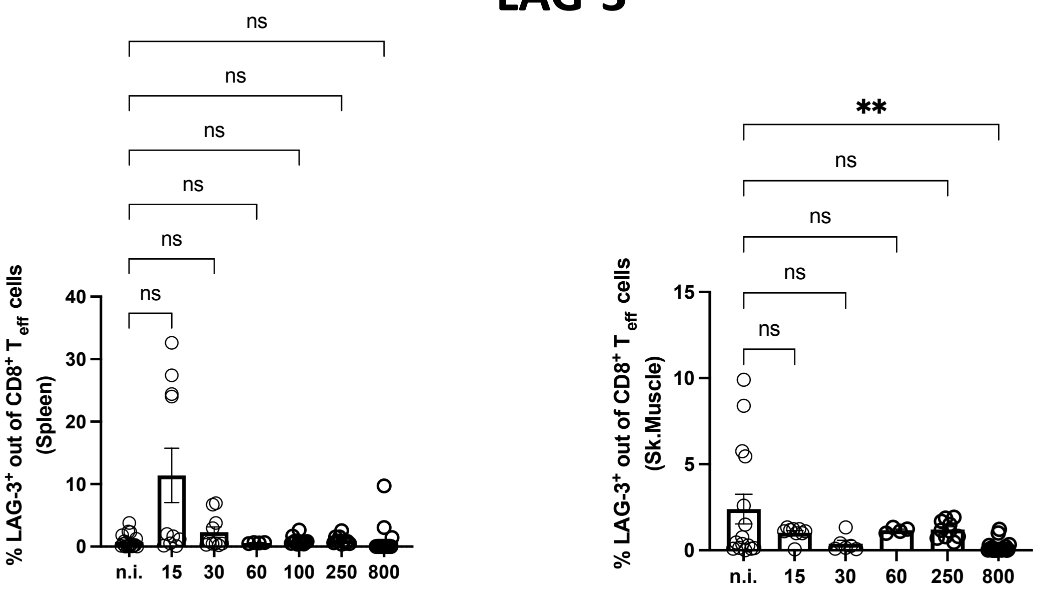

S2. Gating Strategy and exemplary dot blots

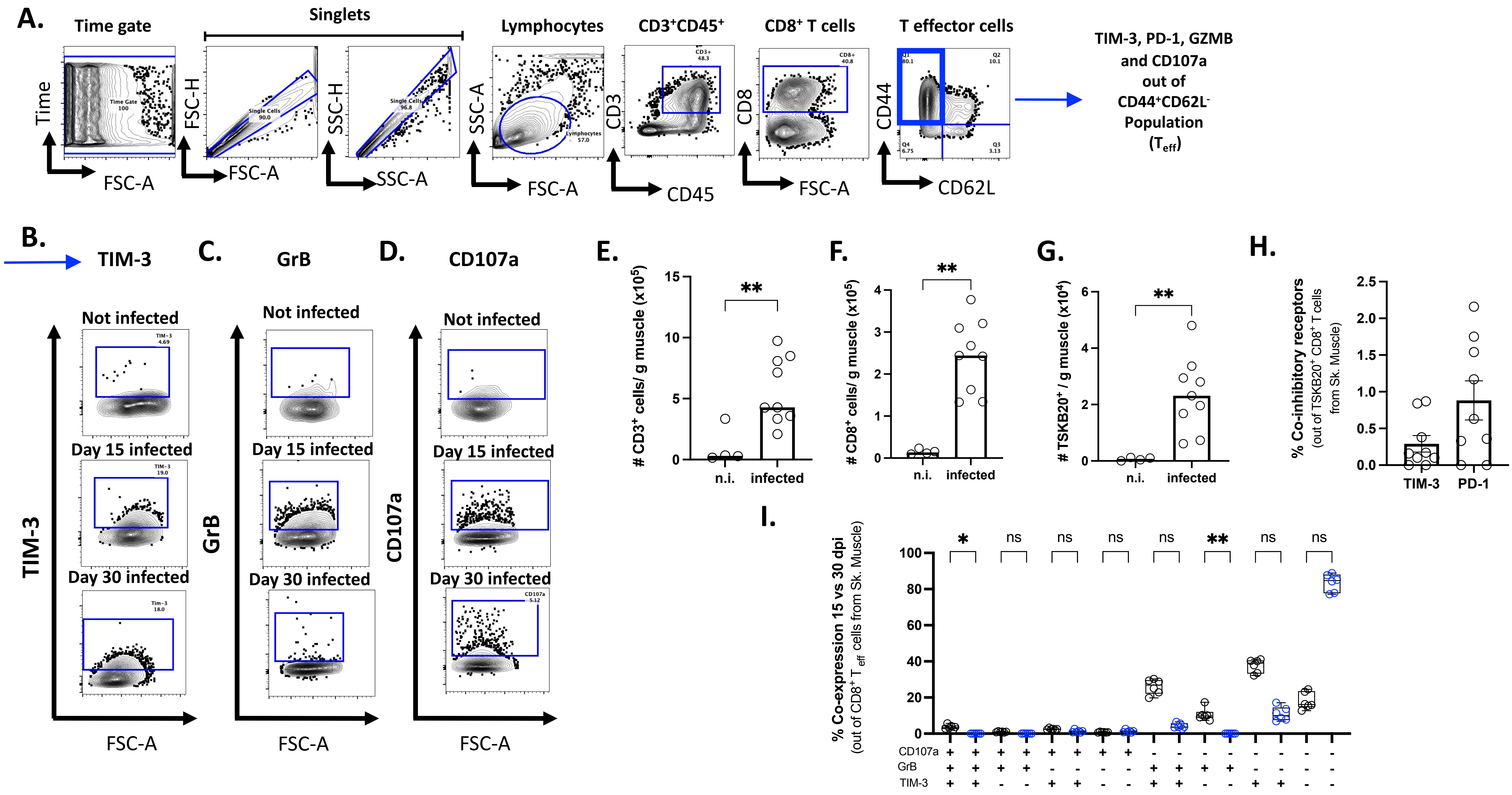

S3. Gating Strategy and exemplary dot blots

A. Not infected

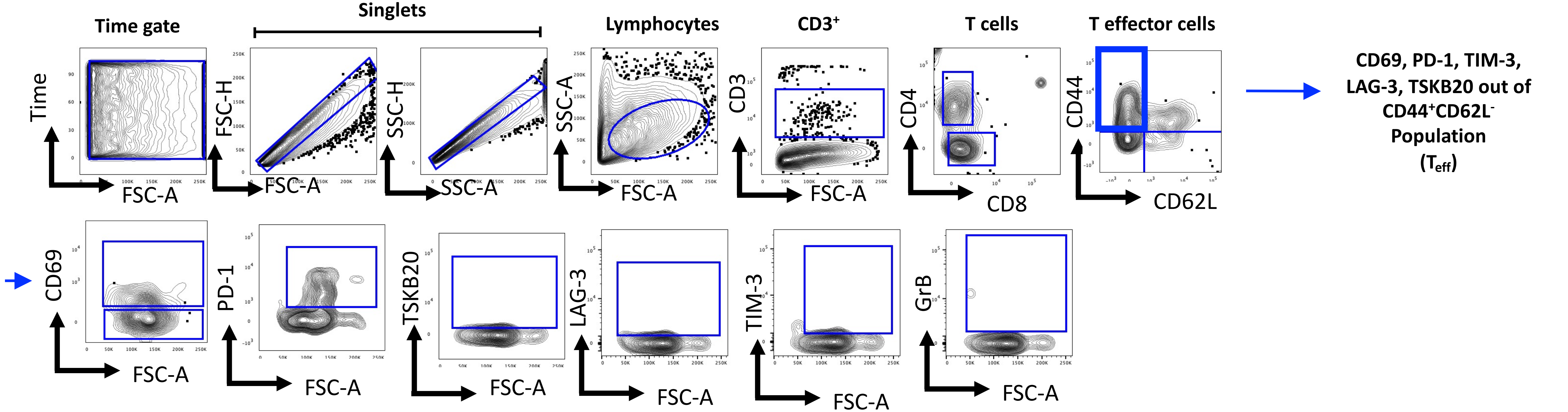

B. Infected

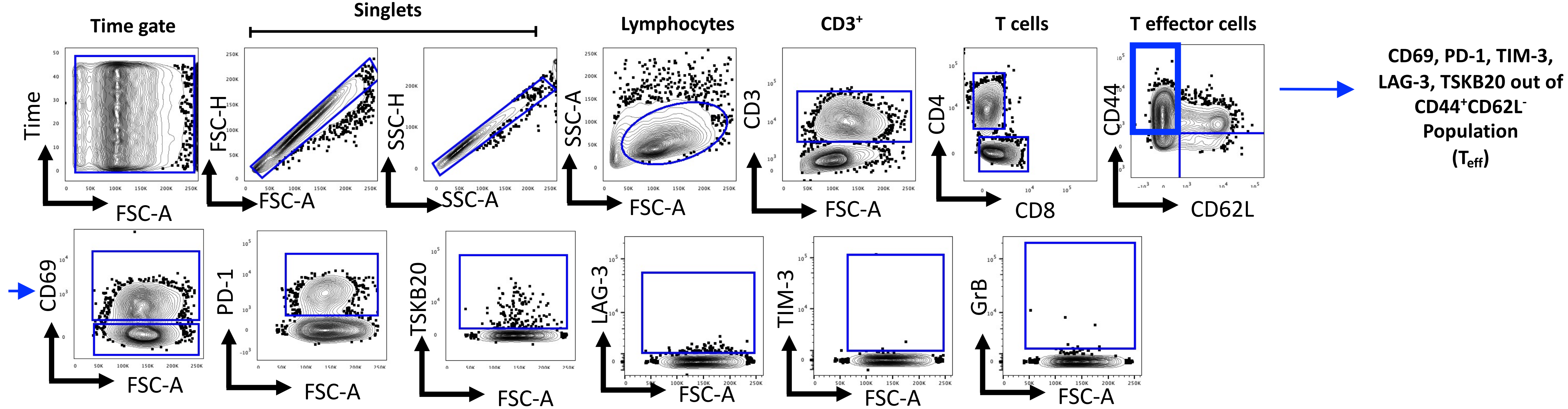

S4.

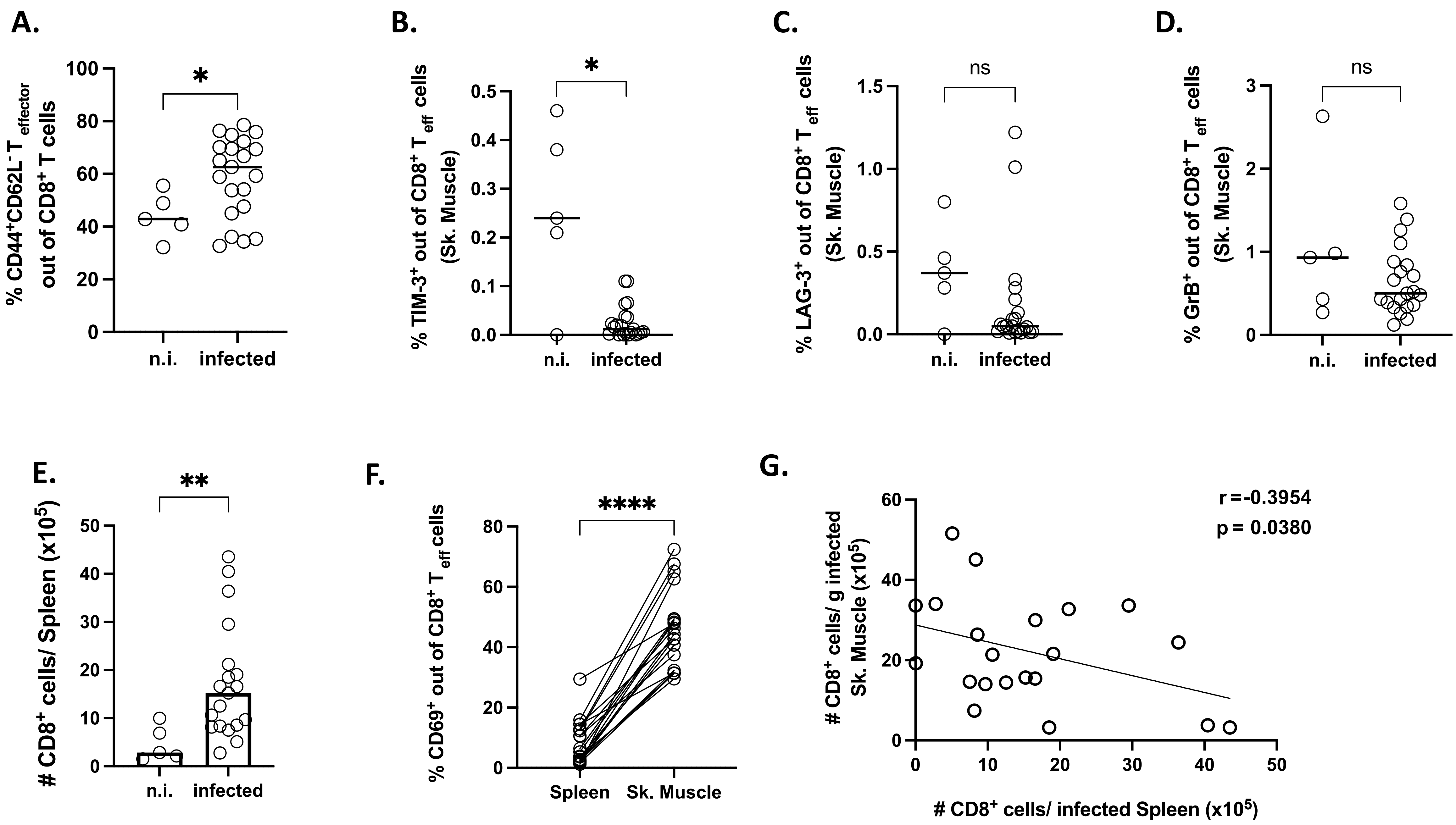
A.
B.
C.
D.
E.
F.
G.
% CD44+CD62L- T effector out of CD8+ T cells
% TIM-3+ out of CD8+ Teff cells (Sk. Muscle)
% LAG-3+ out of CD8+ Teff cells (Sk. Muscle)
% GrB+ out of CD8+ Teff cells (Sk. Muscle)
# CD8+ cells/ Spleen (x10^5)
% CD69+ out of CD8+ Teff cells
# CD8+ cells/ g infected Sk. Muscle (x10^5)
# CD8+ cells/ infected Spleen (x10^5)
n.i.
infected
Spleen
Sk. Muscle
*
**
****
ns
r = -0.3954
p = 0.0380

S5.

A.

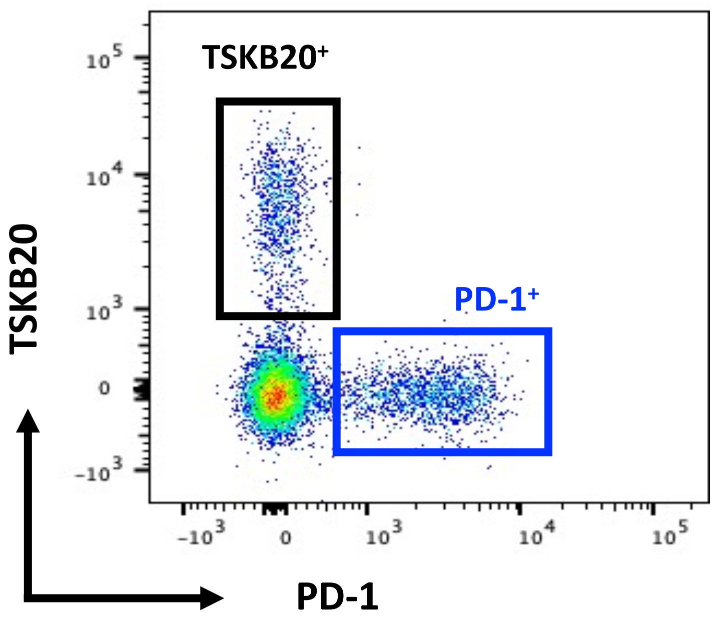

B.

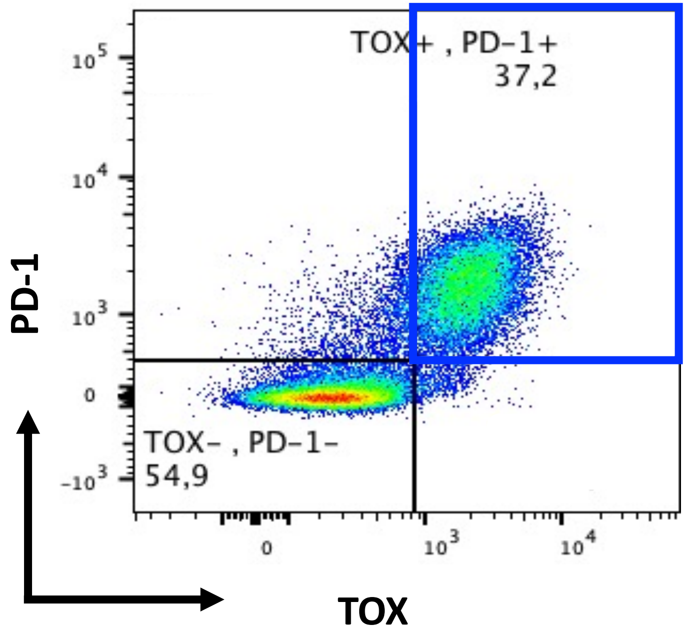

S6. Stimulation of T cells derived from muscles of infected CD45.2 mice *with T. cruzi* lysate-pulsed splenocytes from CD45.1 mice as antigen-presenting cells

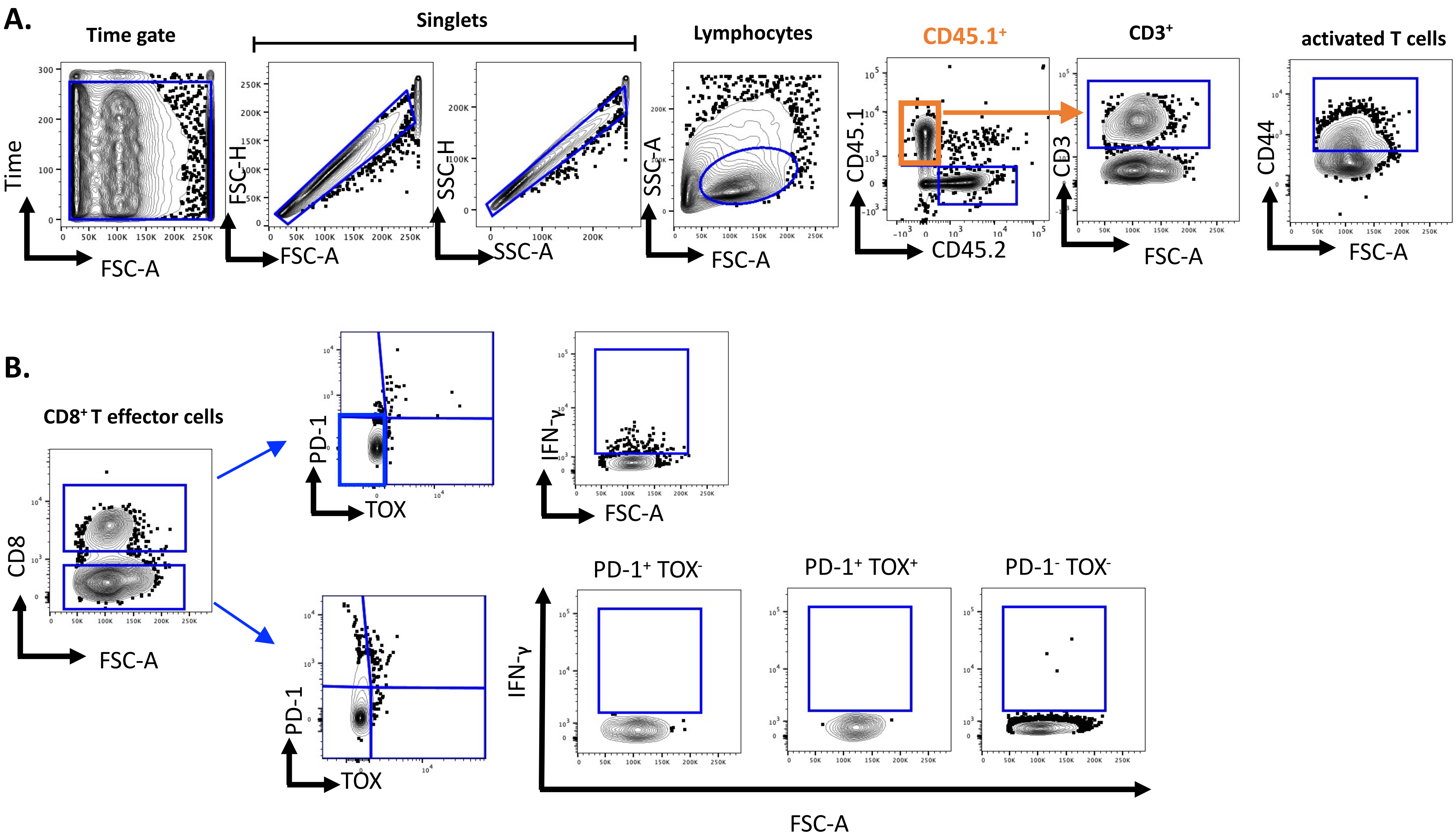

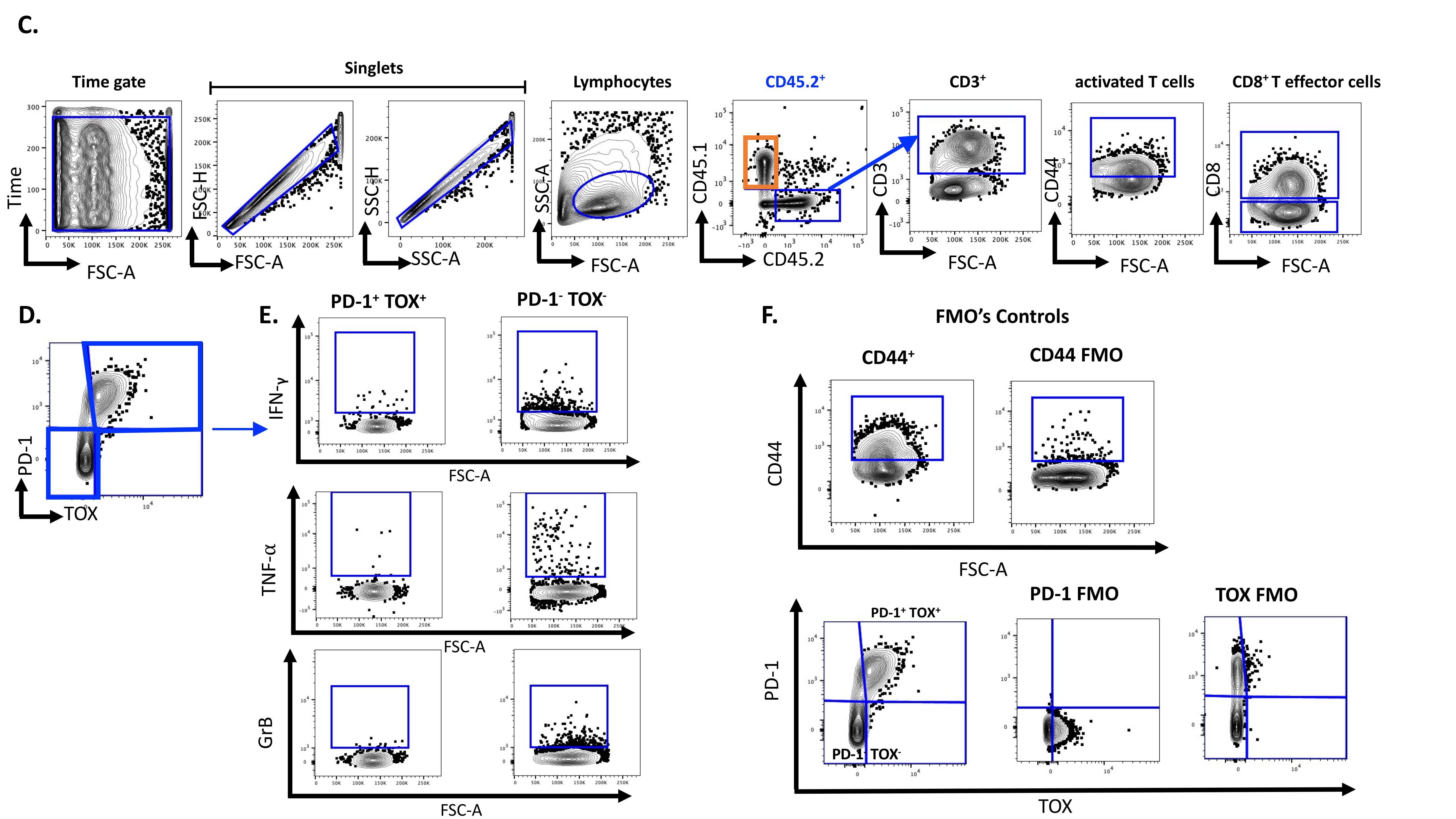
C.
Time gate
Singlets
Lymphocytes
CD45.2+
CD3+
activated T cells
CD8+ T effector cells
Time
FSC-A
FSC-H
SSC-H
SSC-A
CD45.1
CD45.2
CD3
CD44
CD8
D.
PD-1
TOX
E.
PD-1+ TOX+
PD-1- TOX-
IFN-γ
TNF-α
GrB
F.
FMO's Controls
CD44+
CD44 FMO
PD-1 FMO
TOX FMO
PD-1- TOX-
